# Lead-Free Bi_0.5_Na_0.5_TiO_3_ Ferroelectric Nanomaterials for Pyro-Catalytic Dye Pollutant Removal under Cold-Hot Alternation

**DOI:** 10.3390/nano12224091

**Published:** 2022-11-21

**Authors:** Zheng Wu, Siqi Wu, Siqi Hong, Xiaoyu Shi, Di Guo, Yan Zhang, Xiaoli Xu, Zhi Chen, Yanmin Jia

**Affiliations:** 1 Xi’an Key Laboratory of Textile Chemical Engineering Auxiliaries, School of Environmental and Chemical Engineering, Xi’an Polytechnic University, Xi’an 710600, China; 2 School of Science, Xi’an University of Posts and Telecommunications, Xi’an 710121, China; 3 College of Materials and Chemistry, China Jiliang University, Hangzhou 310018, China

**Keywords:** pyro-catalysis, ferroelectric materials, dye pollutant removal, Bi_0.5_Na_0.5_TiO_3_ nanomaterials, pyroelectric materials

## Abstract

In this work, explicitly pyro-catalytic performance is observed in sol-gel-synthesized ferroelectric Bi_0.5_Na_0.5_TiO_3_ lead-free nanomaterials, and its application for dye wastewater purification is also actualized under temperature fluctuations varying from 23 °C to 63 °C. The decomposition ratios of the pyro-catalytic Bi_0.5_Na_0.5_TiO_3_ nanomaterials on Rhodamine B, methyl blue and methyl orange can reach 96.75%, 98.35% and 19.97%, respectively. In the pyro-catalytic process, the probed active species such as hydroxyl radicals, superoxide radicals and holes play an extremely important role in decomposing dye molecules. The ferroelectric Bi_0.5_Na_0.5_TiO_3_ lead-free nanomaterials will have an excellent prospect for dye wastewater purification due to its explicit pyro-catalysis.

## 1. Introduction

In recent years, with the increasing demand for color in textiles and printing, the textile industry has developed rapidly [1,2,3,4,5]. Numerous organic dyes sneak into the water, causing serious water pollution and arousing people’s concern [6,7]. To change this situation, numerous methods have continuously been discovered for purifying dye wastewater [8,9,10,11,12,13], in which photocatalysis technology, as an advanced oxidation method, has caused a great sensation in recent decades [14]. In the photocatalytic process, photo-induced electrons and holes will participate in the chemical reactions to form active species with strong oxidation abilities such as hydroxyl radicals (•OH) and superoxide anions ($•O_{2}^{-})$ for dye wastewater purification [15,16]. Some photocatalysts with excellent dye decomposition performance have been reported, such as ZnO [17] and TiO_2_ [18]. However, the commercial photocatalyst is restricted to some extent, mainly owing to the narrow response range of the visible light wavelength and the low utilization efficiency of solar energy [19]. Therefore, it is essential to seek new catalytic technology for dye wastewater purification.

Apart from sunlight, temperature fluctuations are also an extremely common phenomenon and deemed a readily available energy source [20]. Pyroelectric materials possess the ability to convert thermal energy into electrical energy in the case of temperature fluctuations [21,22,23,24,25,26,27]. Theoretically speaking, it is possible to use the pyroelectrically induced negative and positive electric charges to generate strongly active species and catalytically decompose dye molecules [28,29,30,31,32,33,34,35,36,37]. Recently, some reported materials such as ZnO [38], BaTiO_3_ [39] and NaNbO_3_ [40] can exhibit highly pyro-catalytic activity for purifying dye wastewater, which means that a newly catalytic method via utilizing the natural energy of cold-hot fluctuation can be developed [41,42,43,44,45,46,47,48,49,50].

In the ferroelectric families, lead-free bismuth sodium titanate (Bi_1-x_Na_x_TiO_3_), a sort of perovskite structure, possesses many different oxidation layers and a superb pyroelectric property near the morphotropic phase boundary (MPB) (x = 0.5) [51,52]. It has been reported that Bi_0.5_Na_0.5_TiO_3_ (BNT) possesses the optimal pyroelectric response with a relatively high Curie temperature of 320 °C [53,54], a pyroelectric coefficient (*p*) of 2.5 × 10^−4^ C·m^−2^·K^−1^ and no toxicity to the environment [55,56]. In addition, nanoscale catalysts often possess excellent catalytic performance, mainly owing to their large surface areas [57]. Therefore, nanoscale BNT is an ideal candidate material to be used to investigate the pyro-catalysis for purifying dye wastewater. Up to now, there has been rare reporting on the pyro-catalytic dye decomposition using the pyroelectric BNT lead-free nanomaterials near the morphotropic phase boundary region as the candidate catalyst.

In this study, strongly pyro-catalytic activity for purifying dye wastewater is achieved in sol-gel-synthesized lead-free ferroelectric BNT nanomaterials under cold-hot fluctuation varying from 23 °C to 63 °C. The pyro-catalytic decomposition ratios of BNT nano-catalysts on Rhodamine B (RhB) dye, methyl blue (MB) dye and methyl orange (MO) dye can reach 96.75%, 98.35% and 19.97%, respectively.

## 2. Materials and Methods

### 2.1. Synthesis of BNT

The BNT nanocatalysts were synthesized using a sol-gel method. All the raw materials were of analytical grades. Bismuth nitrate pentahydrate (Bi(NO_3_)_3_·5H_2_O) and sodium acetate (CH_3_COONa) were dissolved in an acetic acid (CH_3_COOH) solution. After stirring for 3 h, a stoichiometric tetrabutyl titanate (Ti(C_4_H_9_O)_4_) solution was added to form a complex sol. Subsequently, the deionized water was appended to the mixed solution (the molar ratio of deionized water to titanium solution was 50:1). Ethylene glycol ether (CH_3_OCH_2_CH_2_OH) was added to stabilize the Ti(C_4_H_9_O)_4_. Then, the PH value of the solution was adjusted to two. The mixture was heated at 55 °C to form a clear and stable precursor. The synthesized precursor was transferred to a 120 °C oven to dry. The dried gel was heated and kept at 650 °C for 1 h.

### 2.2. Characterization

The crystalline structures of the synthesized BNT were characterized on an X-ray diffraction (XRD, Philips PW3040/60, Eindhoven, The Netherlands) using monochromatic Cu Kα radiation (*λ* = 1.5406 Å, 2*θ* = 20°–80°). The surface morphology was characterized by scanning electron microscopy (SEM, Phenom ProX desktop, Alemlo, The Netherlands). The morphology of the BNT was further observed with a transmission electron microscope (TEM, JEOL 2010F, Japan) with an acceleration voltage of 200 kV. The elements in the BNT were analyzed through energy-dispersive X-ray spectroscopy (EDS, Phenom proX, Alemlo, The Netherlands). The ferroelectric hysteresis loop was measured at room temperature through employing a ferroelectric analyzer (RTI-Multiferroic-4 kV, Radiant, Albuquerque, NM, USA) at 100 Hz. To characterize the chemical element composition in the BNT sample, X-ray photoelectron spectroscopy (XPS, ESCALAB 250Xi, Waltham, MA, USA) analysis was performed through using a Thermo Fisher Scientific Ka in wide scan survey mode and a high-energy resolution with Al Ka. All the binding energies were calibrated through using a C 1*s* peak at 284.8 eV of the surface adventitious carbon as a reference.

### 2.3. Pyro-Catalysis Performance Test

These solutions of RhB, MO and MB were selected for testing the pyro-catalytic activities. First, 50 mL of RhB dye (5 mg/L), MO dye (5 mg/L) and MB dye (5 mg/L) solutions were put into three 100-mL glass beakers, respectively. The BNT nano-powders were evenly dispersed in the three types of dye solutions. Before the cold-hot fluctuation, each dye solution with BNT was mechanically stirred for 1 h to achieve the adsorption-desorption equilibrium between the dye solution and catalyst. Here, a water bath was used to achieve uniform cold-hot fluctuation varying from 23 °C to 63 °C in the dark to avoid photodecomposition. Again, every 10 cold-hot cycles, a 2-mL dye solution sample was collected and centrifuged to separate the dye solution and catalyst. The absorption spectra of these dye solutions were recorded through a Hitachi U-3900 (Japan) UV–Vis spectrophotometer.

### 2.4. Detection of Pyro-Catalytic Active Species

The trapping experiments were designed to make certain the active species during the pyro-catalytic process. Iso-propanol (IPA, •OH scavenger) [58], p-benzoquinone (BQ, $•O_{2}^{-}$ scavenger) and ethylenediaminetetraacetate (EDTA, a quencher of holes) were selected as inhibitors [59,60]. The RhB was selected as the experimental dye for trapping of the active species. In the process of the experiment, 2 mL of the dye solution was collected every 10 cold-hot cycles for UV–Vis spectrophotometer measurement.

## 3. Results and Discussion

**Figure 1:**
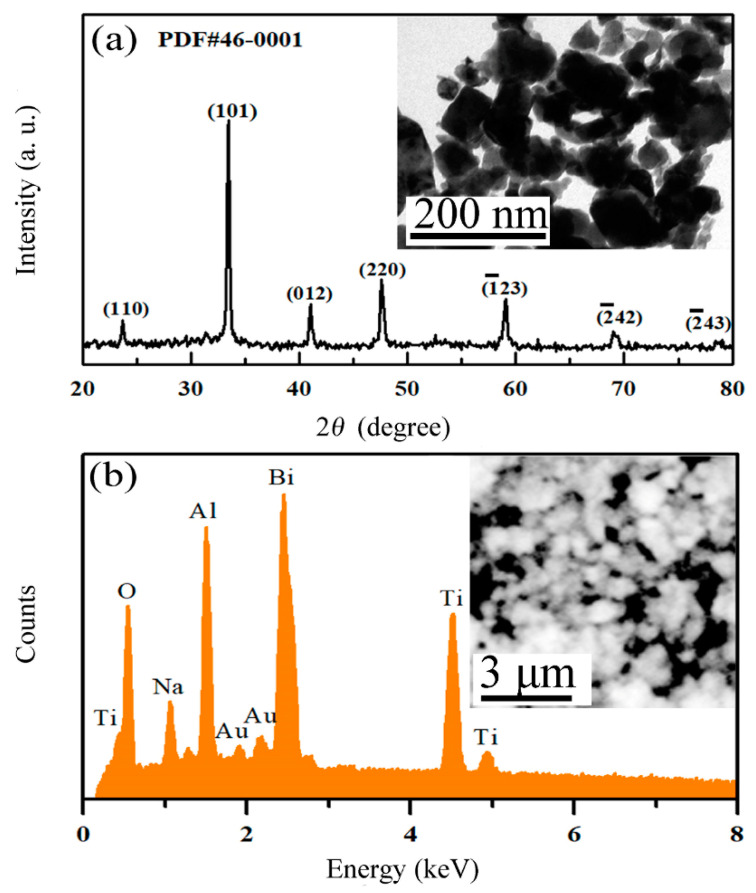
Microstructure characterization of BNT. (**a**) The XRD pattern. The inset is the TEM image. (**b**) EDX analysis. The inset is the SEM image.

Figure 1 shows the microstructure characterization of BNT. Figure 1a depicts the XRD of the synthesized BNT nanopowders, in which the characteristic peaks of 22.8°, 32.6°, 40.2°, 46.7°, 58.2°, 68.4° and 77.8° correspond to the characteristic planes (110), (101), (012), (220), $\left(\bar{1}23\right)$, $\left(\bar{2}42\right)$ and $\left(\bar{2}43\right)$ of monoclinic phase BNT (PDF card number 46-0001), respectively. All characteristic peaks could be observed in the diffractogram, confirming the high purity of the BNT. The TEM image of BNT can be presented through the inset of Figure 1a, which shows that the BNT powders were on the nanometer scale of ~100 nm with a polygonal shape. The EDX image of BNT in Figure 1b clearly confirms the presence of Na, O, Ti and Bi, where the probed Au and Al resulted from the sputtered Au coating on the powder sample and sample holder, respectively. The SEM image is illustrated in the inset of Figure 1b, in which the BNT powder samples show smooth edges and flat surfaces with a well-defined polygonal morphology.

**Figure 2:**
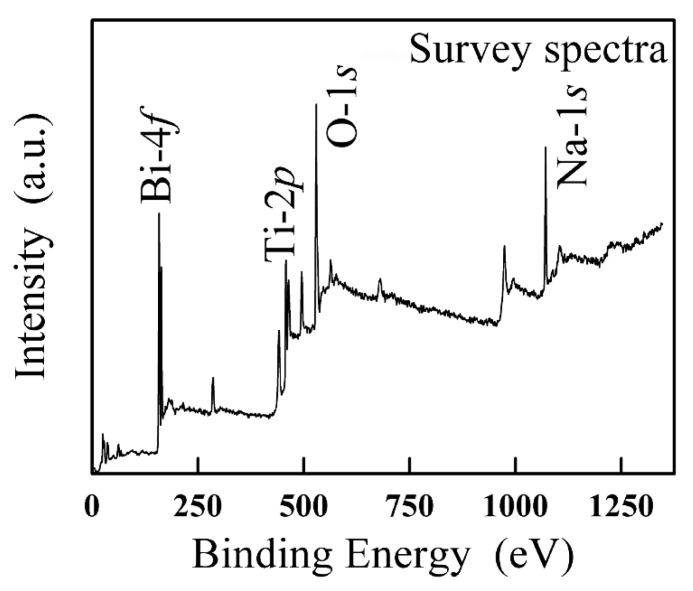
The XPS spectra of BNT.

The survey XPS spectra of BNT is illustrated in Figure 2, in which these peaks of Bi 4*f*, Na 1*s*, Ti 2*p* and O 1*s* for BNT can be intuitively observed. These unmarked peaks correspond with these elemental oxidation states.

**Figure 3:**
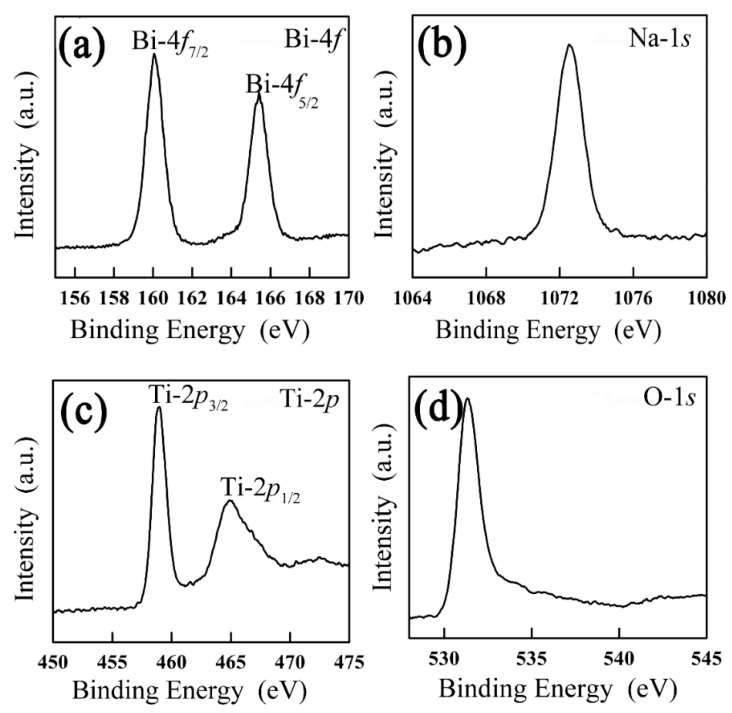
The XPS spectra of BNT: (**a**) Bi 4*f*, (**b**) Na 1*s*, (**c**) Ti 2*p* and (**d**) O 1*s*.

Figure 3 further confirms results of the local magnification of XPS. In Figure 3a, the Bi 4*f* spectrum originated from two contributions, 4*f*_7/2_ and 4*f*_5/2_, located at 160.6 eV and 165.9 eV, respectively. The Bi 4*f* spectrum clearly proves the presence of two chemical environments for Bi atoms. The two strong peaks are allocated to the Bi 4*f*_7/2_ and Bi 4*f*_5/2_ peaks of Bi^3+^ [61], respectively. In Figure 3b, The Na 1*s* peak was detected at 1072.93 eV. The peak corresponds to the Na^+^ cation [62]. In Figure 3c, the Ti 2*p* spectrum also originated from two contributions, Ti 2*p*_3/2_ and Ti 2*p*_1/2_, located at 459.24 eV and 465.34 eV, respectively [63]. Figure 3d shows the binding energy value of O 1*s*. The O 1*s* peak was detected at 531.32 eV.

**Figure 4:**
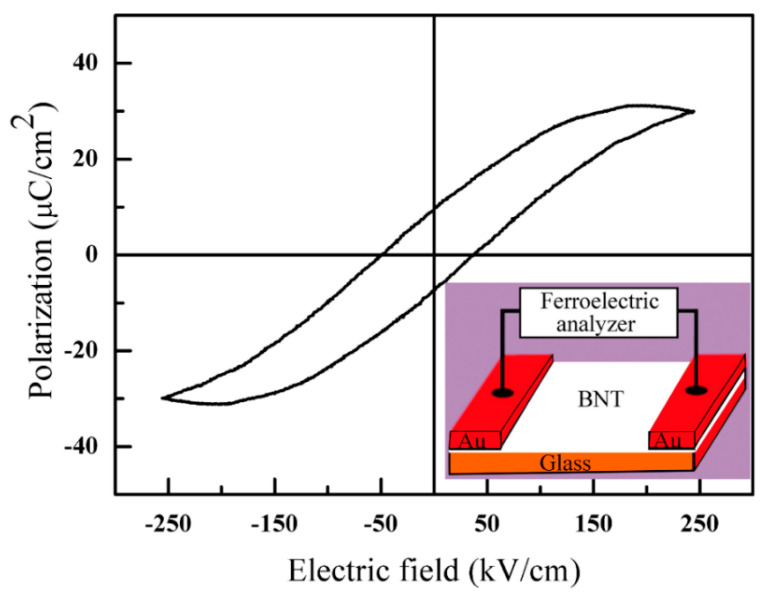
The ferroelectric hysteresis loop of BNT. The inset is the schematic diagram for the ferroelectric measurement.

The ferroelectric loop of BNT can be seen in Figure 4, in which the well-saturated loop indicates the ferroelectric characterization of BNT. The inset of Figure 4 demonstrates the schematic diagram of the circuit for the ferroelectric measurement, with the BNT laid on an insulated glass substrate and the two electrodes made of Au. The pyroelectric properties of the BNT materials were expected, since all the ferroelectric materials were pyroelectric [64].

**Figure 5:**
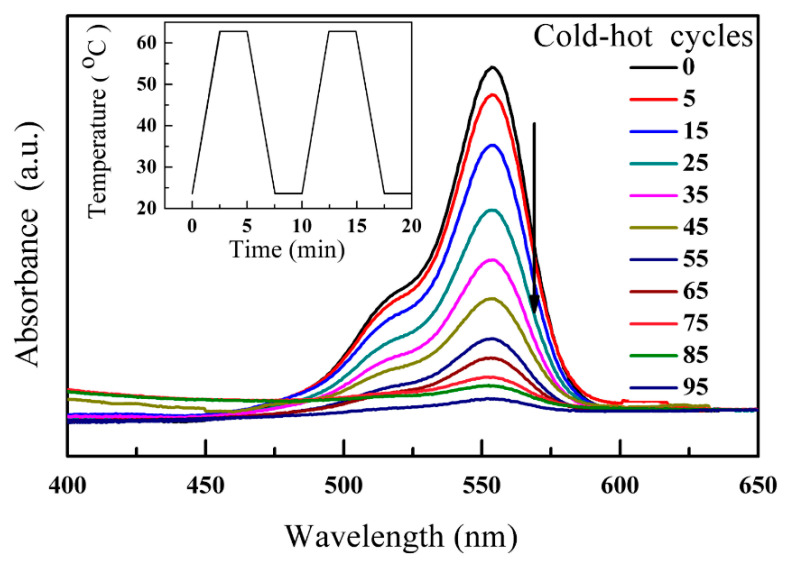
The adsorption spectrum of RhB dye solution with BNT. The inset shows the time curve for the cold-hot cycle.

Figure 5 shows the adsorption spectra of the RhB dye solution with BNT under temperature fluctuations varying from 23 °C to 63 °C. The inset of Figure 5 shows the designed temperature fluctuation curve (from 23 °C to 63 °C) for cold-hot cycles during the pyro-catalytic process. Every dye had its own maximum absorption peak intensity. The absorption peak intensity of the RhB dye was located at ~554 nm. With the increase in cold-hot cycles, the absorption peak height of the RhB dye solution gradually decreased, indicating the excellent pyro-catalytic activities of BNT.

**Figure 6:**
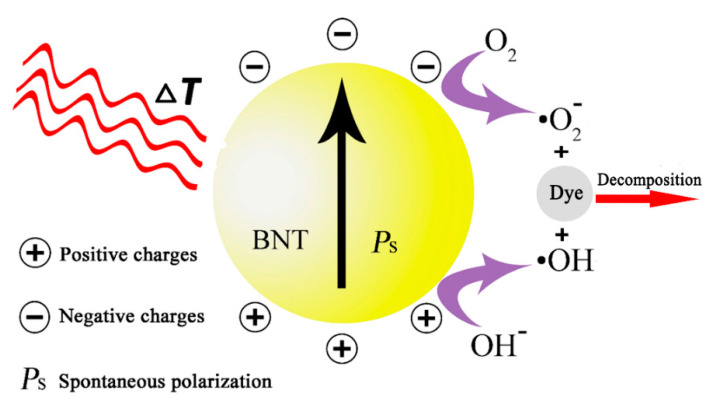
The mechanism diagram of the pyro-catalytic dye decomposition with BNT.

Figure 6 depicts the mechanism schematic diagram of pyro-catalysis under the cold-hot fluctuation. When the temperature is altered, these positive and negative charges will be generated on the surfaces of the pyroelectric BNT catalysts based on Equation (1) [65,66]:

(1) $$\mathrm{BNT}\overset{\Delta T}{\to }\mathrm{BNT}+q^{+}+q^{-}$$

The extra electric charges be equipoised by the charge compensation of $O_{2}$ and ${\mathrm{OH}}^{-}$ in a water solution. These negative charges ($q^{-}$) will produce the activated superoxide radicals (•$O_{2}^{-}$) based on Equation (2):

(2) $$O_{2}+q^{-}\;\to \;•O_{2}^{-}$$

Similarly, the positive charges ($q^{+}$) will produce the hydroxyl radicals (•OH) based on Equation (3):

(3) $${\mathrm{OH}}^{-}+q^{+}\to \;•\mathrm{OH}$$

Finally, these active species decompose stubborn dye molecules based on Equation (4):

(4) $$•\mathrm{OH}\;\mathrm{or}\;•O_{2}^{-}+\mathrm{Dyes}\to \mathrm{Dye}\;\mathrm{decomposition}$$

**Figure 7:**
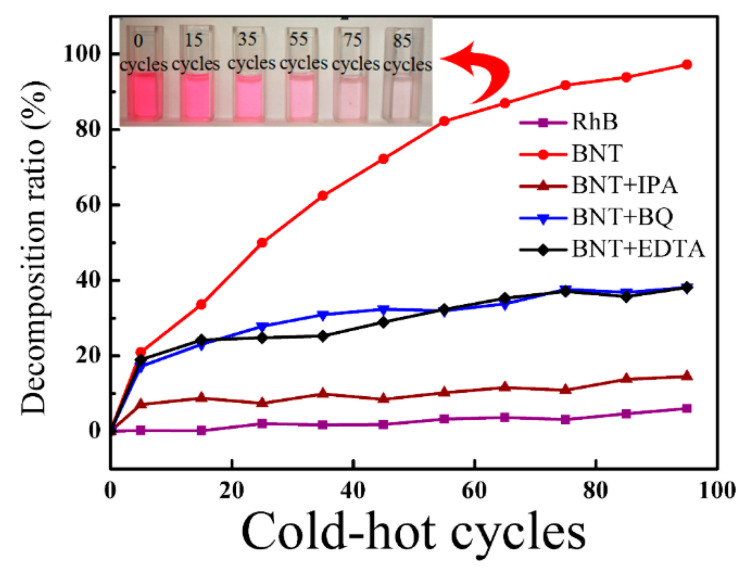
The pyro-catalytic decomposition ratio of RhB and inhibitory effect of different inhibitors on BNT. The inset shows these pictures of RhB dye decomposition.

Figure 7 presents the results of the decomposition ratio of RhB dye under different conditions and the surface radical trapping experiments, in which the decomposition ratio (*D*) of the RhB dye can be calculated as *D* = (1 − *C*/*C*_0_), where *C*_0_ (*C*_0_ = 5 mg/L) and *C* are the initial concentration and the actual concentration of the dye solution during the pyro-catalytic process, respectively. In Figure 7, the decomposition of the RhB dye was significantly inhibited by IPA, and the addition of EDTA and BQ was also heavily inhibited, Furthermore, the color change of the RhB dye solution with BNT is shown in the inset of Figure 7, in which the color of the RhB dye solution became transparent after 85 cold-hot cycles. These results indicate that RhB dye is difficult to self-decompose under temperature fluctuations, the decomposition behavior of RhB dye results from the pyro-catalysis, and the extreme importance of •OH is shown, similarly indicating that $•O_{2}^{-}$ and holes are the main active species in the pyro-catalytic process.

**Figure 8:**
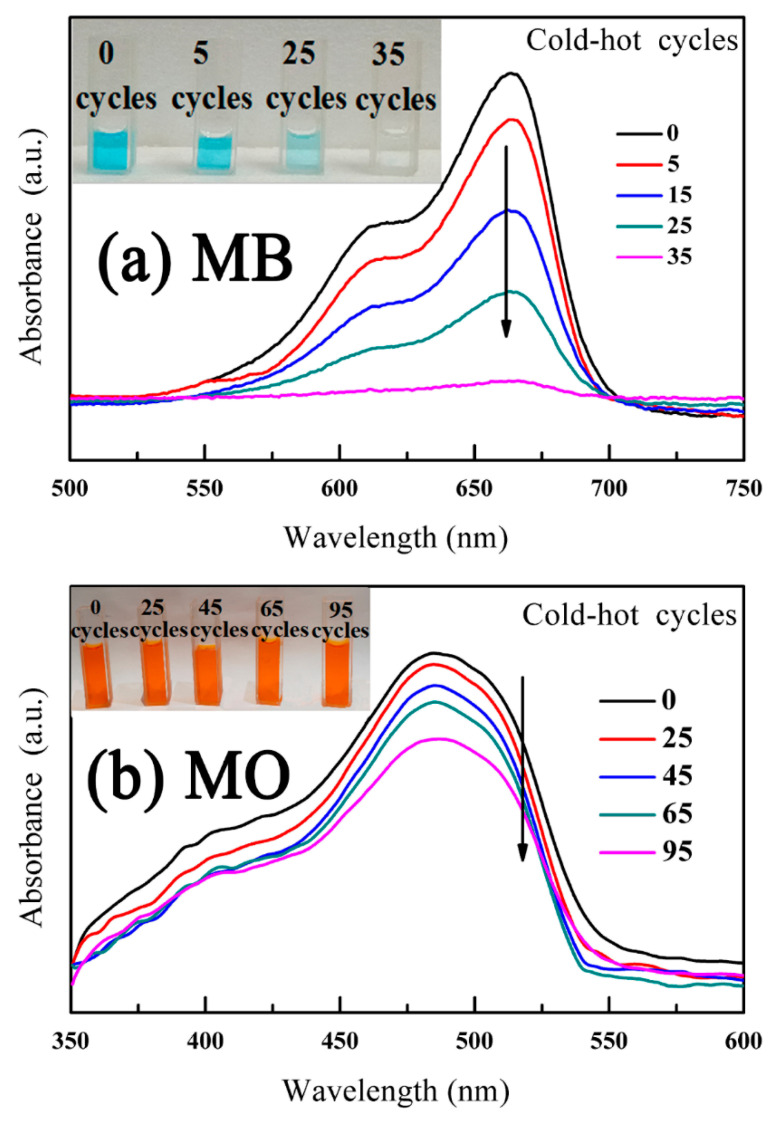
The characterization of pyro-catalysis performance of BNT: (**a**) MB and (**b**) MO dye solutions. The inset shows pictures of MB or MO dye decomposition.

Figure 8 shows the decomposition results of the MB dye and MO dye, in which the adsorption spectra of the MB and MO solutions correspond to Figure 8a,b, respectively. The absorption peak intensities of the MB dye and MO dye are located at ~664 nm and ~485 nm, respectively. With the increase in cold-hot cycles, the absorption peak heights of the two dyes gradually decreased. Furthermore, the color changes of the MB dye and MO dye are exhibited in the inset of Figure 8a,b, respectively. The color in the MB dye solution became transparent after 35 cold-hot cycles, while the color in the MO dye solution changed to almost nothing after 85 cold-hot cycles.

**Figure 9:**
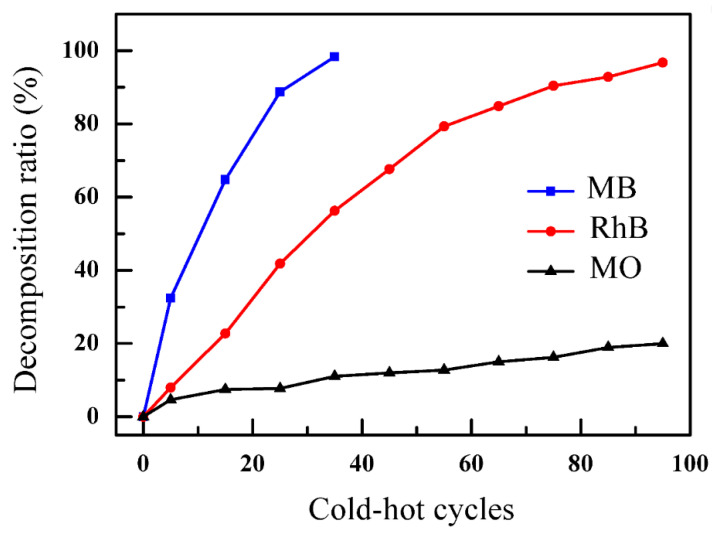
The pyro-catalytic dye decomposition ratios for different dye solutions.

The pyro-catalytic activities of BNT for different dye solutions are reflected through the decomposition ratios in Figure 9. The decomposition ratios of the RhB dye and MO dye solution reached 96.75% and 19.97%, respectively, after undergoing 95 cold-hot cycles, while the decomposition ratio of the MB solution was 98.35% after undergoing 35 cold-hot cycles. These different decomposition activities may be associated with their organic molecular chain structures [67], which cause different decomposition mechanisms. In theory, decomposition reactions begin to occur between a dye molecule and a strong oxidation radical [68]. The decomposition process was accelerated, owing to the de-ethylating reaction in the RhB dye solution, causing high pyro-catalytic activity [69,70]. During the radical reactions, the sulfur located on the aromatic links in the MB dye molecules was more reactive than the sulfonyl group in the *p*-benzene ring of the MO dye molecules. Additionally, the molecules of MB and RhB are much larger in size than those of the MO dye, which is conducive to being absorbed and broken [71]. As shown in Figure 9, the decomposition ratio of the MO dye was the lowest.

In addition to the pyro-catalytic activity exhibited in this work, Li et al. have reported that the BNT nano-catalyst also possesses remarkable photocatalytic performance, with a ~91.6% decomposition ratio for MO dye [72]. Recently, a practical method named light-thermal synergy to further enhance catalytic decomposition ratio was reported [73]. D. Jiang et al. have reported that the catalytic dye decomposition ratio through thermo-or photo-bi-catalysis synergy is further enhanced compared with that of pyro-catalysis or photocatalysis itself [74]. Therefore, the amelioration of catalytic activities in BNT may be expectable, through the synergy of thermo- or photo-bi-catalysis in the future [75,76,77].

## 4. Conclusions or Summary

In summary, a remarkable pyro-catalytic phenomenon for dye wastewater purification was observed in sol-gel-synthesized BNT ferroelectric lead-free nanomaterials under cold-hot fluctuation varying from 23 °C to 63 °C. The pyro-catalytic decomposition ratios of the BNT nanomaterials on RhB dye, MB dye and MO dye were 96.75%, 98.35% and 19.97%, respectively. The catalysis reaction on the BNT catalysts’ surfaces under the environmental temperature fluctuation was excited by these pyroelectrically induced active species with strong oxidation abilities. This promising pyro-catalysis in lead-free BNT ferroelectric nanomaterials has potential in purifying dye wastewater utilizing cold-hot fluctuations.

## Data Availability

The data that support the findings of this study are available from the first author or corresponding authors upon reasonable request.
